# Some Facts Relative to Diseases of the Teeth and Jaws in Inebriety

**Published:** 1893-04

**Authors:** T. D. Crothers

**Affiliations:** Hartford, Conn.


### Some Facts Relative to Diseases of the Teeth and Jaws in Inebriety.

BY T. D. CROTHERS, M.D., OF HARTFORD, CONN.

Read in the Section of Oral and Dental Surgery, at the Forty-third Annual Meeting of the American Medical Association, held at Detroit, Mich., June, 1892.

Seen from the clinical and neurological standpoint, a large proportion of the hereditary cases of inebriety have defects and degenerations of the face, jaws and teeth.

Up to the present time, no studies have indicated any *specific* degeneration that can be traced to alcohol as the sole cause. Nevertheless, the inebriate from heredity or other causes has defects, malformations and retarded growths of various organs of the body that are hints of continued organic degeneration. The inebriety or any associated disease in the parents may appear very prominently in defects of the face, head and jaws. These widely differing variations from the normal symmetries are almost infinite in diversity and degree. The same variations in quality and capacity exist in the brain.

How far the bony structure of the calvarium is controlled by the brain itself or how far external defects influence the internal development, is not yet clearly understood.

The frequency with which congenital defects of the head and face appear in inebriates suggests a pathological relation, which is fully in accord with other facts, and are also true of other degenerative processes. The complex degenerations from the excessive use of alcohol are always transmitted in some form to the children.

Low vitality and defective cell growth and nutrition appear in the descendants in aggravated forms.

The children of such parents are always noted for physical defects of structure and type, although they may display widely varying degrees of brain power—from genius to idiocy.

Inebriety is often only developmental disease, beginning in embryonic life, and manifest in various degrees of malformations. A child with organic defects at birth is switched on the road to inebriety, insanity and perhaps idiocy. Harelip, cleft or deformed palate, talipes, rickets and other abnormalities are unmistakable hints.

A child in infancy who has convulsions at dentition, strabismus, and later chorea, epilepsy, asthma, who at puberty suffers from hysteria, arrested growths, stupidity, or abnormal sensitiveness is on the same road with the same destination.

If to this is added malformed maxillaries and palate arches, together with defective teeth, the evidence is complete.

It is an important question, however, how far a deformed palate or defective teeth can be accepted as evidence of neurotic or alcoholic diathesis. If the question is confined to the teeth, some curious facts appear.

It may be said to be an established fact that the teeth of hereditary neurotics vary in shape and structure from those of persons of different heredity. Probably they are not any more marked in many cases than any other organic degenerations. Some of these defects do not appear in organic form or structure, but in the power and quality of force displayed.

The effect of alcohol in excess on the teeth is within the observation of all persons. This may be described in general terms to be that of an irritant, impairing the vitality of the mucous membranes and developing a grade of chronic inflammation that is continuous. The stomach, throat, and mouth all become the seat of degenerative processes, by which the nerves of the teeth are quickly affected. The nutrition of the teeth is impaired and the pulp dies, the gums retract, often leaving a red line of demarkation.

The effect of alcohol on the system is to paralyze and impoverish the nutritive system. Conditions of practical starvation supervene; affecting all growth. Hence, degenerate teeth and gums are the natural outcome.

It is a fact that inebriates, as a rule, suffer from early disease and death of the teeth. A large proportion of cases under my care seek the aid of the dentist early in the treatment. When the teeth are destroyed and replaced by others, carelessness in the quality of food, in mastication, and methods of eating are sources of irritation that farther predispose to the use of spirits. The neurotic diathesis that is seen in the early decay of the teeth will, in many cases, develop inebriety, as the result of impoverished brain nutrition.

Irregularity of the teeth is also very prominent in inebriates. Projecting incisors and molars that crowd past each other and various other deformities are common. I have in a few cases noticed what seemed to me a very unusual absorption of the bone where teeth have been withdrawn, leaving great cavities, usually seen only in advanced age. This has appeared to me to be a part of the customary alcoholic degeneration.

Some years ago a very large, broad shouldered, muscular inebriate came under my care. He had a thin, piping voice. Examination revealed a very high, narrow palate arch, with a projecting lower maxillary. He was an hereditary neurotic from an insane and moderate drinking family. His father had the same high vaulted palate, but a retreating, imperfectly developed lower maxillary. He had two brothers, one a famous tenor singer, with a striking astigmatic face, who no doubt had a high palate arch. The second had a high arch with a flat dome and irregular teeth and jaws.

Within a year I have seen two children of this patient, both with deformed palate arches and teeth. They are markedly neurotic and have strongly marked degenerative heredity.

It is a common observation that a thin, high-pitched voice is a possible hint of hereditary degeneration when seen in inebriates, and it is often associated with deformed mouth or maxillaries.

This observation of Dr. Talbot appears to be amply confirmed in my experience. Reasoning from the general history of these cases, I should expect to find many deformed palates and teeth, and other marks of physical defects, such as cranial deformities, facial irregularities, together with ill-shaped hands, feet, arms and legs.

Retarded growths and over-growths, associated with mental defects, are often very prominent.

From a clinical point of view, a deformed palate may be taken as an unmistakable sign of neurotic degeneration, which may or may not develop into a distinct form of disease. If with this are associated bad teeth, a tendency to break out into inebriety doubtless exists, due to nerve and cell starvation, with consequent exhaustion, for which alcohol is a most seductive narcotic. In a large per cent. of inebriates, where a deformed palate is found, it can be taken as a sign of nerve and brain degeneration, and will be accompanied by other physical signs of like import. Further study of these cases will reveal mental defects of reason, conduct, and character.

The inebriate who has this peculiar arch with defective maxillary growths has received from his ancestors a pathological bias which will make a cure difficult.

I shall close this brief note with a quotation from Dr. Clausten's work on the "Neuroses of Development":

"If the skull in its growth, its size and shape, its dome and base, is actually dominated by the brain it defends and contains, then the brain growth will in this way secondarily determine the shape of the upper maxillary bone and the palate.

"The brain unquestionably deriving its shape, size, and qualities from ancestry and a bad heredity, determining a bad brain, we see how a bad nervous heredity would naturally determine an abnormal palate. The theory that a high palate may be a "reversion" to a lower animal form or to the form in lower races of mankind seemed to be largely disproved by a careful examination of the palates of most classes of animals and of different races from the Europeans to the extinct Americans, the Australians and the Hottentots, which I made in the Anatomical Museum of Edinburgh University.

Where dolichocephaly and brachiocephaly are a matter of race and not of disease or degeneration, they make no difference in the palate dome.

There is no such thing as a high V-shaped palate in any animal or in any race from the Australian aborigines upward. In one way only can the V-shaped palate be connected with a reversion to the animal type. Apart from prognathism, the sides of the palate, that is, the lines of the bicuspid and molar teeth on each side tend to run parallel to each other.

Now, supposing the lines of the teeth in man during development were approximated and made parallel by a formative reversionary process, the palate would be pushed up in the center—the line of the least resistance—into the nasal cavity.

Those palates where the deformity consisted in a ridge down the center antero-posteriorly, seem to show that in them the deformity took place at a later period than in the other deformed palates when the nasal septum was getting stronger and kept the center of the palate down, while on each side of it the palate was drawn up making two palate vaults instead of one.

The theory that the shape of the palate is dependent on the nasal cavity of which it is the floor seems to me without any valid foundation whatever.

The upper maxillary bone is related to three functions, viz.: alimentation, smell, and facial expression.

There is no proof that either smell or mastication is interfered with by a high dome, but unquestionably facial expression is greatly influenced by any change in its form, and facial expression is naturally related directly to the development of the mental part of the brain and is dependent on it. Speech I found is affected slightly by the shape of the palate.

The theory that deformed palates are due to thumb-sucking in infancy is utterly baseless. Idiots notoriously don't suck well.

Prognathous jaws certainly seem to be a reversion to an animal and lower human type, but they do not necessarily imply a high palate.

Virchow's theory of premature or irregular ossification of the skull sutures—though it may explain some few cases of idiocy—does not seem to me to explain the high palate any more than I think it explains the dolichocephalic or brachiocephalic types of head or the types of cerebral sulci.

The deformity of the palate occurs during brain growth, early in life, probably in utero.

We must refer the high palate to a bad initial neurotic heredity, just as we refer a bad type of face or irregular teeth or an asymmetrical head to such heredity.

Mere accidental changes in the head shape are quite consistent with good brain development. The brain in its growth, if artificially confined in one direction will expand in another, and not suffer in function thereby.

The savages who make their children's heads square by pressure do not alter the mental or motor functions of the brain thereby.

The vaulted palates and altered dental arch must be taken with other changes in the head and especially in the face-expression, as one of the morphological indications that show a tendency in the person to whom it belongs and in his family to developmental neurotic diseases—notably idiocy, congenital imbecility, stuntedness of growth, deformity, epilepsy, adolescent insanity, and that organic lawlessness and lack of mental inhibition or weakness of mind that distinguish the criminal classes. It is therefore one of the marks of a family that is tending toward mental death and extinction.

As regards the exact mode in which the palate deformity arises, I cannot find any help in a study of the mode of ossification of the upper maxillary bone, except in the cases where deformity consists in a depression or two depressions where the four centres of ossification meet. If the anterior lobes of the brain—and that is the part of the brain that lies above the palate—become contracted through hereditary brain deficiency, and the skull base in front is therefore narrowed while the jaw must remain large enough to hold a normal number of teeth, it might naturally assume more the shape of the bow of a boat than a horse-shoe.

The alveoli of the bicuspids and molars on each side being drawn together by the narrow skull base from which they hang this process would throw up and deform the palate.

Clayshaw's measurements of the skull-base and pterygoid arch give much support to this hypothesis.

In congenital mental defect it is the anterior lobes that are found chiefly deficient, and in the microcephalic and Kalmuck class of idiots—where the brain has undergone most developmental lessening, the palate is found to be highest and most deformed.

Taking all the facts into account, it seems proved that the condition of the palate may be a most important index of brain development and of liability to developmental neurosis.

My second class of neurotic palates is apt to go with a nervous temperament—often with a neurotic diathesis; often with high mental qualities, but not all-roundness of capacity; often with keen sensitiveness or supersensitiveness of bodily feeling or emotion, and frequently with liability to the lesser functional neuroses—such as hysteria, neuralgia, migraines, headaches, etc.

It seemed to me that the consumptives are more apt to have neurotic and, many of them, deformed palates, than the sound in body.

I did not find any connection, hereditarily or otherwise, between my "deformed" palate and cleft palate.

They seem in a way to represent formative tendencies "in utero" in opposite directions.

In the case of the ossifying centres in cleft palates they had not been able to meet in the center and close in the floor of the nares, through perhaps excessive width of skull base; while in the latter, as we have seen, there was a greater width of palate bone than was needed to stretch across, and so it had to rise up in the center and form an acute angle or high arch.

### DISCUSSION.

Dr. J. Taft said the most remarkable effects noted in the paper were on the shape of the jaw and mouth due to hereditary transmission. These effects were important and undeniable, but the question arises whether the inebriate suffered any change in the conformation of these parts induced by the habit of drinking.

Dr. Crothers said there were very marked changes, but chiefly those resembling the changes usually brought about in the process of aging.

Dr. Barrett said he understood the essayist to refer to the V-shaped vault as a reversion to the animal type. He thought this was a mistake, as the high or V-shaped vault is not found among animals. It, however, might be true in regard to the V-shaped arch, as this, or rather the saddle-shaped arch, is common in animals, more especially those nearest correlated to man.

Dr. E. S. Talbot: I am much interested in the paper of Dr. Crothers, especially as the author's long connection with an inebriate asylum gives him peculiar advantages for observing these conditions. I will have to take issue, however, with his statements that these peculiarities of the mouth and jaws extend back to uterine life. Every observing dentist knows better than that. The author being a medical man, has not the opportunity for observing the mouths of all classes which we of the dental profession have. We know that this condition is never found during the existence of the first set of teeth. The deformity begins in the sixth or seventh year.

For several years I have been making a study of these peculiarities in the mouths of idiots, mutes, inebriates and criminals. In that time I have examined about seven hundred and fifty mouths of inebriates, and have found a larger percentage of badly formed mouths than among any criminal, idiot, or other defective class; but the defects are not so marked. There are fewer absolutely V-shaped arches among inebriates, but more of what might be called partial V-shaped arches; but I find plenty of V-shaped arches among criminals, idiots, the deaf and the blind. Now another singular thing: There are twice as many high vaults among inebriates as among any other class of degenerates. This proves that the inebriate is born; that it is impossible for him to live a straight, ordinary life. One born with the qualities that this formation indicates will become either an extreme egotist, a genius in business or some other direction, or he will be deaf and dumb, idiotic, blind, or an inebriate. I will show this in a paper I am to read before the Neurological Section, and will show that the inebriate is diseased.

If we examine a skull we find quite a space between the hard palate and the brain. If the brain is arrested in its development, the osseous system will likewise be undeveloped, but the arrest of development in the osseous system will be caused by the want of development in the brain. I may say, however, that the lower jaw is developed independently of the rest of the face.

Dr. Fletcher asked whether the fact that both the teeth and the brain were developed from the epiblast had any bearing upon the defective teeth in the classes spoken of.

Dr. Talbot thought that as the arrest of development was from causes back of the birth of the child, it was reasonable to suppose that there was a defect in the tissue from which the organs originated.

